# Genetic evaluations of Chinese patients with odontohypophosphatasia resulting from heterozygosity for mutations in the tissue-non-specific alkaline phosphatase gene

**DOI:** 10.18632/oncotarget.18093

**Published:** 2017-05-23

**Authors:** Jia Wan, Li Zhang, Tang Liu, Yewei Wang

**Affiliations:** ^1^ Department of Orthopaedics, The Second Xiangya Hospital of Central South University, Changsha 410011, P.R.China; ^2^ Department of Endocrinology, The Fifth Central Hospital of Tianjin, Tianjin 300192, P.R.China; ^3^ Department of Hemotology, The Second Xiangya Hospital of Central South University, Changsha 410011, P.R.China

**Keywords:** hypophosphatasia, odonto-hypophosphatasia, gene mutation, premature exfoliation of primary teeth, TNSALP

## Abstract

**Background:**

Hypophosphatasia is a rare heritable metabolic disorder characterized by defective bone and tooth mineralization accompanied by a deficiency of tissue-non-specific (liver/bone/kidney) isoenzyme of alkaline phosphatase activity, caused by a number of loss-of-function mutations in the alkaline phosphatase liver type gene. We seek to explore the clinical manifestations and identify the mutations associated with the disease in a Chinese odonto- hypophosphatasia family.

**Results:**

The proband and his younger brother affected with premature loss of primary teeth at their 2-year-old. They have mild abnormal serum alkaline phosphatase and 25-hydroxy vitamin D values, but the serum alkaline phosphatase activity of their father, mother and grandmother, who showed no clinical symptoms of hypophosphatasia, was exhibited significant decreased. In addition to premature loss of primary teeth, the proband and his younger brother showed low bone mineral density, X-rays showed that they had slight metaphyseal osteoporosis changes, but no additional skeletal abnormalities. Deoxyribonucleic acid sequencing and analysis revealed a single nucleotide polymorphism c.787T>C (p.Y263H) in exon 7 and/or a novel mutation c.-92C>T located at 5’UTR were found in the affected individuals.

**Materials and Methods:**

We examined all individuals of an odonto- hypophosphatasia family by clinical and radiographic examinations as well as laboratory assays. Furthermore, all 12 exons and the exon-intron boundaries of the alkaline phosphatase liver type gene were amplified and directly sequenced for further analysis and screened for mutations.

**Conclusion:**

Our present findings suggest the single nucleotide polymorphism c.787T>C and c.-92C>T should be responsible for the odonto- hypophosphatasia disorders in this family.

## INTRODUCTION

**Figure 1:**
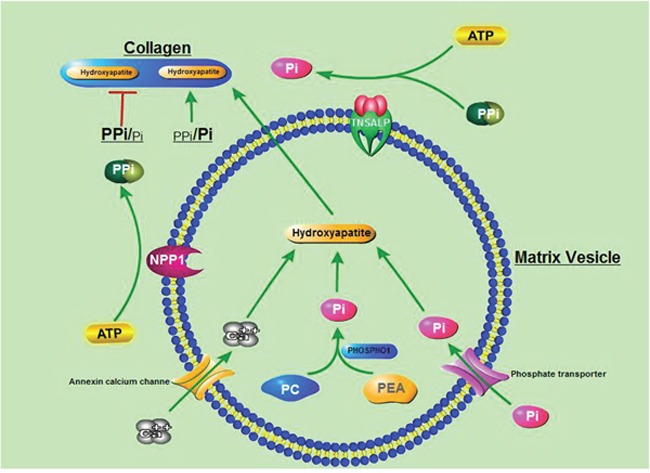
Schematic illustration of the process of mineralization Mineralization begins with hydroxyapatite formation in the matrix vesicles budding from hypertrophic chondrocytes and osteoblasts. Hydroxyapatite is formed from Ca^2+^ incorporated by the annexin calcium channel and from Pi. The source of Pi involves two independent biochemical pathways: intravesicular Pi generation by the enzymatic action of PHOSPHO1 and influx of Pi, generated in the perivesicular space by the activities of TNAP and NPP1, via phosphate transporter. PPi which inhibits hydroxyapatite formation, is hydrolyzed by TNAP. The balance between the PPi and Pi is crucial for the mineralization. PC: phosphocholine; PEA: phosphoethanolamine; Pi: inorganic phosphate; PPi: inorganic pyrophosphate.

Hypophosphatasia (HPP) is a rare inherited metabolic disease characterized by defective bone and tooth mineralization and a deficiency of tissue-non-specific (liver/bone/kidney) isoenzyme of alkaline phosphatase (TNSALP) activity. The disease is caused by mutations in the alkaline phosphatase liver type (ALPL) gene [1]. The symptoms of this disease are vary widely in clinical manifestation, which spans from stillbirth without skeletal mineralization in utero or at birth to premature loss of teeth without bone symptoms occuring among childhood or adult life [2]. Six clinical categories of HPP have been classified according to the age at diagnosis and the severity of the symptoms: lethal perinatal, benign perinatal, infantile, childhood, adult, and odonto-hypophosphatasia (odonto-HPP) forms [3, 4]. The ALPL gene, localized on chromosome 1p36.1-34, comprises of 12 exons scattered over 50 kilobases [5, 6]. Thus far, at least 335 mutations are known and reported in the tissue nonspecific alkaline phosphatase gene mutations database (ALPL gene mutation database). TNSALP hydrolyzes mineralization inhibitor, inorganic pyrophosphate (PPi), and provides inorganic phosphate(Pi) to promote the process of mineralization. When TNSALP activity is reduced or lost, extracellular PPi accumulates and inhibits the formation of hydroxyapatite [7, 8]. Consequently, the inhibition of hydroxyapatite formation leads to deficient mineralization of bones and teeth accompanied by highly variable clinical manifestations (Figure 1) [9].

Odonto-HPP is the least severe of HPP characterized by premature exfoliation of primary teeth and/or severe dental caries as an isolated finding without additional abnormalities of the skeletal system or as part of the other forms of HPP [10–12]. Dental X-rays shows reduced alveolar bone, enlarged pulp chambers and root canals [11]. Biochemical findings are essentially indistinguishable from those of patients with mild forms of childhood or adult HPP [2].

We report two brothers with premature exfoliation of primary teeth as their only clinical manifestation of HPP.

## RESULTS

### Clinical examination

**Figure 2:**
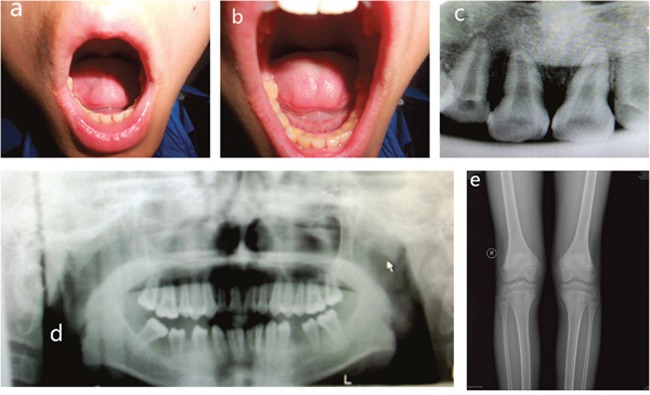
Clinical features of proband **(A, B)** Intra-oral photograph of proband showing that permanent teeth were erupted. **(C, D)** Panoramic radiographs showing normal permanent teeth. **(E)** Plain films of a knee joint presenting with metaphyseal osteoporosis changes.

**Figure 3:**
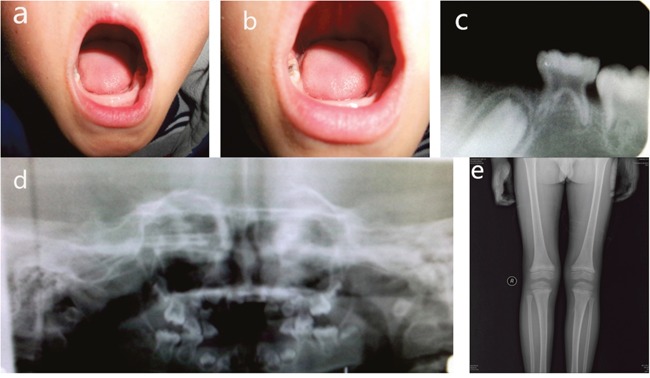
Clinical features of younger brother **(A, B)** Intra-oral photograph of proband showing premature exfoliation of primary teeth. **(C, D)** Panoramic radiographs showing reduced alveolar bone, enlarged pulp chambers, and abnormal morphology of the unerupted permanent molars. **(E)** Plain films of a knee joint presenting with metaphyseal osteoporosis changes.

Panoramic radiographs of the proband demonstrated that the erupted teeth were normal and the radiographs of the younger brother showed reduced alveolar bone, enlarged pulp chambers, and abnormal morphology of the unerupted permanent molars. X-ray of knee joints presented metaphyseal osteoporosis changes in femurs and tibiae (Figures 2 and 3). Ultrasonographic assessment of BMD in distal radius and middle tibia showed low bone mineral density for the proband and his younger brother. In addition, their levels of serum ALP moderately decreased, whose values were 41.5 U/L and 43.4 U/L, respectively (regular scope for children 110-550 U/L). No aberrant variation in serum Ca, PTH, ESR and microelement were detected. But a high level of serum P (2.08 mmol/L and 1.97 mmol/L, respectively, normal range for children 0.90-1.34 mmol/L) and low serum 25-hydroxy vitamin D (50.40 nmol/L and 55.70 nmol/L, respectively, normal range for children 75-175mmol/L) were observed.

The serum alkaline phosphatase (ALP) levels of the father, mother and grandmother were below normal, which is 25.4 U/L, 41.9U/L and 25 U/L, respectively (normal range for adult men 62-176 U/L, adult women 55-147 U/L). Their serum electrolyte level was normal, but serum 25-hydroxy vitamin D level was slightly decreased. However, they all had no history of precocious absent of primary teeth and bone diseases. The other individual of the proband's family (grandfather) was healthy, and neither clinical signs nor laboratory assays detected any abnormal (Table 1).

**Table 1:** Clinical Characteristics of the pedigree in this study

	Age(years)	Premature loss of teeth	Serum ALP (U/L)	Serum 25-hydroxy vitamin D (nmol/L)	Serum Ca (mmol/L)	Serum P(mmol/L)	Parathyroid hormone (PTH) (pmol/L)
Grandfather	68	No	Normal	Normal	Normal	Normal	Normal
Grandmother	65	No	25(55-147)	65.3(75-250)	Normal	Normal	Normal
Father	40	No	25.4(62-176)	70.4(75-250)	Normal	Normal	Normal
Mother	38	No	41.9(55-14)	68.5(75-250)	Normal	Normal	Normal
Son #1	14	Yes	41.5(110-550)	50.4(75-250)	Normal	2.08(0.90-1.34)	Normal
Son #2	6	Yes	43.4(110-550)	55.7(75-250)	Normal	1.97(0.90-1.34)	Normal

### Mutation analyses

**Figure 4:**
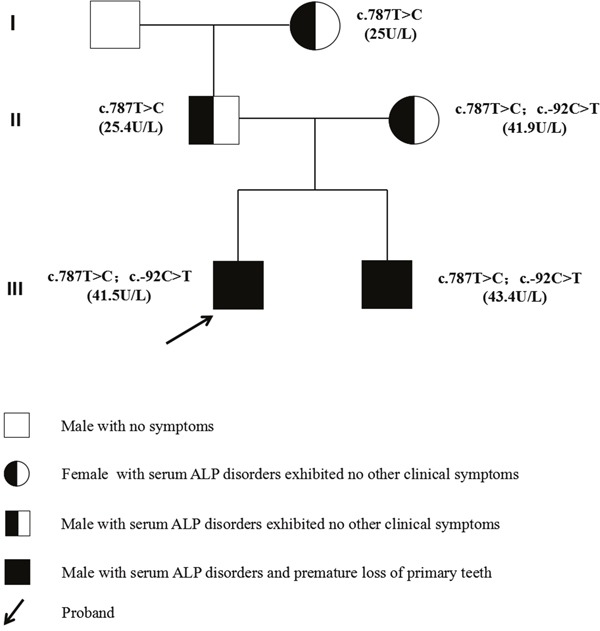
Kindred pedigree Three generations depicting which women or men carry the TNSALP mutation.

**Figure 5:**
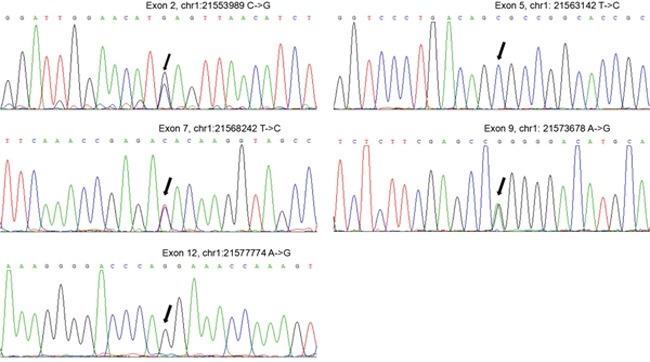
Genetic analysis of the HPP family Sequencing chromatographs of genomic DNA from the proband; the arrow indicates the position of the mutation.

Pedigree data and mutation analysis results are shown in Figure 4. Screening for mutations in the ALPL gene disclosed five hereditary change in the brothers. Firstly, there was a heterozygous substitution T to C at nucleotide position 787 (c.787T>C) in exon 7, it leads to a tyrosine to histidine substitution at the 263th amino acid (p.Y263H). The second and third alterations identified in the brothers were mutations in the 5’ untranslated region (5’-UTR) c.-92C>T and 3’ untranslated region (3’UTR) c.1701A>G. The fourth and fifth were non-sense mutations, located in exon 5 (c.330T>C) and exon 9 (c.876A>G), respectively. Other family members were also analyzed the ALPL genes, and the results showed that all four adults were carriers of genetic alterations [the father: c.787T>C (p.Y263H), c.T330T>C (non-sense), c.876A>G (non-sense) and 3’UTR (c.1701A>G), the mother: 5’UTR (c.-92C>T), c.787T>C (p.Y263H) and c.876A>G (non-sense), the grandfather: c.330T>C (non-sense) and 3’UTR (c.1701A>G), the grandmother: c.787T>C (p.Y263H) and c.876A>G (non-sense)]. Of the five mutations identified, 4 are single nucleotide polymorphisms (SNPs). These SNPs were: c.330>TC, c.1701A>G, c.876A>G and c.1701A>G. Only the mutation of c.-92C>T located at 5’UTR is absent from the SNP database and the ALPL gene mutation database, thus, these patients (the two brothers and their mother) carry a new missense mutation in the ALPL gene. In addition, the SNP c.787T>C (p.Y263H) was present in a heterozygous state, and this mutation was detected in the family members exhibiting a decrease in serum ALP levels, which confirmed that the sequence aberration is closely related to the serum ALP disorders in this family (Table 2 and Figure 5).

**Table 2:** Mutation analysis of the pedigree in this study

	Exon 1	Exon 2	Exon 3	Exon 4	Exon 5	Exon 6	Exon 7	Exon 8	Exon 9	Exon 10	Exon 11	Exon 12
GF	wt	wt	wt	wt	SNP	wt	wt	wt	wt	wt	wt	SNP
GM		wt					SNP		SNP			
Father	wt	wt	wt	wt	SNP	wt	SNP	wt	SNP	wt	wt	SNP
Mother		mut					SNP		SNP			
Son #1	wt	mut	wt	wt	SNP	wt	SNP	wt	SNP	wt	wt	SNP
Son #2	wt	mut	wt	wt	SNP	wt	SNP	wt	SNP	wt	wt	SNP
Genomic location		Chr1:21553989C-> G			Chr1:21563142T->C		Chr1:21568242T->C		Chr1:21573678A->G			Chr1:21577774A->G
DNA location		c.C-92>G			c.T330C		c.T787C		c.A876G			c.A1701>G
Protein location		5′UTR			Non-sense		p.Y263H		Non-sense			3′UTR

Wt: wild type; mut: mutation; SNP: the variants can be find in the SNP database; GF: grandfather; GM: grandmother.

## DISCUSSION

HPP is a rare hereditary disorder of bone and teeth metabolism caused by loss of function within ALPL gene encoding TNSALP [1]. Human TNSALP protein is a chain of 524 amino acid residues, HPP derived from any mutation in the ALPL gene resulting in a decrease in the efficient activity of TNSALP and leading to extracellular accumulation of PPi, a TNSALP substrate and potent inhibitor of mineralization [13, 14]. The high clinical variability of the HPP is associated with considerable large number of allelic heterogeneity of the ALPL gene. To date, 335 mutations have been found in the ALPL gene, which are associated with HPP. About 71.6% of the known mutations are missense mutations, and the remaining including small deletions accounted for 11.0%, splicing mutations accounted for 6.0%, nonsense mutations accounted for 4.8%, small insertions accounted for 3.0%, large deletions accounted for 2.7%, insertions or deletions accounted for 0.96% and and regulatory sequence mutations accounted for 0.3 %.

Odonto-HPP refers to dental manifestations alone occurring in HPP, usually independent of the additional abnormalities of the skeletal system [15]. The odonto-HPP, due to the mild of symptoms or as part of the other forms of HPP, is not well documented. Only a few mutations responsible for odonto-HPP have been published compared to other forms of HPP. In the present study, our sequencing analysis results showed that a heterozygous T to C transition at c.787 in exon 7, which leading to a tyrosine to histidine substitution at position 263 in the affected members. The unaffected members of the analyzed family did not carry the same mutation. The ALPL gene mutation database also did not contain the mutation information, but this mutation can find in the SNP database. These findings altogether support that the c.787T>C (p.Y263H) is a pathogenic mutation in the ALPL gene. Therefore, we believe that the SNP (c.787T>C) is the cause of the serum ALP disorders in this family. Furthermore, the SNP c.787T>C that a tyrosine to histidine substitution at position 263 was previously shown to decrease the bone mineral density (BMD) and catalytic property of TNSALP in postmenopausal Japanese women, in which the *K*m value was decreased significantly. [16, 17]. However, another study in favor of the sequence variation in linkage disequilibrium (LD) with haplotype E have an aggravating factor resulting in loss of haplosufficiency in European ancestry HPP patients, rather than supporting the role of c.787T>C polymorphism itself [18]. The TNSALP protein is a homodimeric phosphomonoesterase linked to the plasma membrane via a glycosylphosphatidylinositol(GPI) ancchors attached to its carboxyl terminus [11, 19]. The enzyme is activated as a homodimer rather than a monomer [9]. Because of the structural properties of the TNSALP, some ALPL gene mutations that affect protein structures may exhibit dominant negative effects in heterozygotes, leading to mild HPP phenotypes. These mutations exhibited dominant negative effect by inhibiting the enzymatic activity of the wild-type (WT) in heterodimers [20, 21] or mutated protein sequestrate the WT protein into the cell and prevents it from being transported to the membrane [20]. The mutant carriers may not show the disease clearly and promote the variability of the clinical phenotype of HPP. Therefore, we suggest that SNP c.787T> C may show a dominant negative effect, leading to a variable clinical phenotype of the family.

A novel mutation, c.-92C>T, located at 5’UTR was identified in the brothers and their mother in this study. However, the clinical symptoms and signs of odonto-HPP is special in affected individuals with c.-92C>T mutation of this family, the brothers' serum ALP is lower than normal and exhibited significant premature loss of primary teeth, but their mother with below normal serum ALP level exhibited no other clinical symptoms related to HPP. Although the mother show no typical clinical manifestations of odonto-HPP (tooth loss), these results suggest that the heterozygosity of c.-92C>T may be associated with clinical symptoms in this family, but the disease is expressed at least in the mother. And we propose that the proband and his younger brother feature an c.-92C>T missense mutation of maternal inheritance in the regulatory sequence, which does not change the amino acid sequence but may promote the proband and his brother to show more aggressive dental phenotypes.

In conclusion, we identified the SNP c.787T>C in the ALPL gene in a Chinese family with hypophosphatasia. This SNP may interfere with ALP activity, leading to a decrease in serum ALP levels. In addition to this SNP, the novel mutation c.-92C>T located at 5’UTR may lead to more serious dental phenotype with the SNP c.787T>C (p.Y263H). Our findings will further improve the knowledge of the relationship between regulatory sequence and phenotype, and have benefits to preimplantation genetic diagnosis and prenatal diagnosis. Further study will assess the effect of 5'UTR mutations on protein function.

## MATERIALS AND METHODS

### Patients

Two Chinese brothers, aged 6 and 14 years, were presented with premature exfoliation of primary teeth during early childhood life. Otherwise, they reported good health without evidence of bone disease. Their parents were asymptomatic and non-consanguineous.

The 14-year-old male proband due to early loss of primary teeth, was referred to our hospital in June 2014. He began to lose his teeth at the age of 2, and lost all his teeth when he was 5 years old. However, after suffering a period of agomphosis malformation, his permanent teeth gradually began to erupt around 8 years old, and so far 26 permanent teeth have appeared. Moreover, the proband had no evidence of bone manifestations. The proband's younger brother, a 6-year-old boy, also appeared premature loss of primary teeth, so far has lost 12 primary teeth. The most frequent lost teeth are the incisors. Moreover, the little boy exhibited slight short stature and pectus excavatum.

In order to study the possibility of suffering from HPP, two boys conducted a further examination. The alveolar bone, pulp cavity, root and root absorbance were performed by panoramic X-ray examination. The bone mineral density (BMD) was measured by the quantitative ultrasonography. The X-ray of knee was performed to detect bone growth and pathological changes. The next laboratory tests included serum ALP, Ca, P, 25-hydroxyvitamin D, parathyroid hormone (PTH), erythrocyte sedimentation rate (ESR) and microelement.

### Family studies

Subsequently, the pedigree of their family was investigated for HPP. Laboratory tests included serum total ALP activity, electrolyte and 25-hydroxy vitamin D of the two boys’ parents and grandparents. None of them reported history of premature loss of primary teeth, fractures, weakness, skeletal pain, or growth retardation.

### Mutation studies

Blood samples of family members were collected under Institutional Review Board-approved protocols, and written informed consent was obtained in accordance with the Declaration of Helsinki. Briefly, total RNA was isolated and extracted from peripheral blood cells using Trizol reagent (Invitrogen, CA, USA). One microgram of total RNA was subjected to reverse transcription with the random hexamers. Polymerase chain reaction (PCR) was then performed using cDNA as the template with the indicated primer pairs. The PCR products were then purified and sequenced directly (ABI 3730; Applied Biosystems). Primer sequence of ALPL gene exons was shown in Table 3.

**Table 3:** Primer sequence of ALPL gene exons

Exon	Primer sequence (5′->3′)	Chromosome position (hg38)
1	F: AAGCCAGATATGTTGACAGAR: GCCATTAAAGTTCAACCA	Chr1: 21508967-21509622
2	F: CTGGTCTGTAATAGGTGCTCACR: GTTTCCTGCTCTGAACACTGT	Chr1: 21553856- 21554276
3-4	F: CTCCAAGTTCAGGCATTCCAGR: CGCAAGCAGGTACAGTGATG	Chr1: 21560516- 21561395
5	F: AGGAAGCAGGCAGCTAGGTAGTR: ATCTCAAGTGGACTGTGGCTCTG	Chr1: 21562927- 21563747
5-6	F: TAGGTAGTCCTGTGGCTCTGGR: CCTGGATGCCTGGTTCTTGG	Chr1: 21562941- 21564460
7	F: GGAAGCCAAGTAAGGTAAGTTATCR: CTCAATGTCCACGCAGGTTAT	Chr1: 21567849- 21568504
8	F: CATTAGAACATCACCTCCACCAGR: GCCTAATTCCAGGAACCAGAAC	Chr1: 21570028- 21570548
9	F: GTCACAGCCTCTCAGCATCCR: CTCCTTCCACAACCTATTCTCCT	Chr1: 21573621- 21573941
10-11	F: CAGGTTGAATGGCTGCGTAAR: CTGCTAGATTGTAGAAGGCGATT	Chr1: 21575556- 21576834
12	F: CAGGCTCAGGTTCAAATCCCR: AATGTTCCACGGAGGCTTCA	Chr1: 21577207- 21578001
